# Design of Building Environment Detection System for Architectures Based on Internet of Things

**DOI:** 10.1155/2022/5438305

**Published:** 2022-03-28

**Authors:** Dongfang Zhang, Hui Ji, Zhennan Li, Hui Ge

**Affiliations:** ^1^School of Planning and Design, Xinyang Agriculture and Forestry University, Xinyang, Henan 464000, China; ^2^School of School of Architecture and Urban Planning, Guangdong University of Technology, Guangzhou, Guangdong 510000, China

## Abstract

In the process of urban building design, a new integrated system for monitoring the environment is developed and designed by using embedded development technology and sensor technology. The system uses a wireless sensor network environment monitoring system IoT platform with embedded internal processors. Analyze and design the system as a whole, including the construction of the basic platform of the system, the design of the internal plates and circuits of the system, the monitoring design of the input node, and the monthly design of the output interface calculation. Finally, a physical model is built, and data measurement and analysis are carried out under different conditions, and the evaluation and advantage analysis of the system's operating status are given. The system can carry out all-round, multilevel, and three-dimensional real-time monitoring of the construction site environment, including dust, *PM2.5*, temperature, humidity, wind speed, carbon dioxide, and other indicators in the construction site environment. In addition, the system can upload various monitoring data to the detection system through the internal network. The system has the functions of monitoring, alarming, recording, querying, and counting of the target monitoring station and can also be linked with the environmental control device. The construction site staff can conduct real-time supervision through the mobile terminal and computer terminal management platform. In addition, it can also meet the role of real-time remote monitoring and online guidance and regulation. It has reference value for the safety and management of the actual operation process of the project.

## 1. Introduction

The construction industry has always been a fast-growing industry. With the emergence of more and more modern high-tech, building environment monitoring system has also become a hot research topic in the field of construction. Before this, people could only passively adapt to the external environment using the materials in the living environment. However, due to the gradual development of science and technology, people have gradually become active in transforming the living environment and have taken active measures to improve the environment of various buildings in terms of building site selection, planning and design, building envelopes, building equipment, and building materials [1]. Most of the office buildings in our country have serious energy consumption, the unit energy consumption is gradually increasing, and its energy expenditure is also increasing. This requires research on a method to reduce the energy consumption of buildings. Although many scholars have studied building energy consumption monitoring systems, there are many energy consumption monitoring points in buildings and complex wiring. In this way, the monitoring effect is not good, and the energy consumption of the building cannot be effectively reduced. Based on this problem, on the basis of the building site environmental monitoring software we mentioned above, we design and regulate a building comprehensive environmental energy consumption monitoring and management system based on the Internet of Things technology to solve the above problems [2]. In the traditional building environment monitoring field, the system usually uses a distributed monitoring system composed of an upper computer and a lower computer to monitor the building environment, and the lower computer and various sensors use network communication. This type of monitoring system has the characteristics of flexible structure, easy expansion and upgrading, and low cost [3]. However, with the continuous development of building intelligence, people's requirements for building environment monitoring are also increasing, which usually requires long-distance remote monitoring and big data analysis and decision-making. Therefore, if the traditional environment monitoring system architecture is still adopted, the disadvantages such as heavy wiring workload, troublesome data processing, and unsuitability for long-distance transmission are prominent. With the continuous development of Internet of Things technology and wireless transmission technology, it is possible to design a wireless building environment monitoring system with little or no wiring workload [4].

The role of the Internet of Things has been used in industry, medical care, agriculture, logistics, transportation, power grid, environmental protection, fire protection, furniture, and other aspects, which closely connected with life. With the continuous development and maturity of the technology, the Internet of Things will bring revolutionary changes to life in the future and help human beings to step into the era of wisdom faster and more stably. With the development and improvement of information technology, sensor technology, and Internet of Things technology, it is possible to build an integrated system suitable for long-term monitoring of building environment. In the actual operation state of the building, the performance data such as the energy consumption of the built environment and the indoor environment and the user satisfaction evaluation are collected, and the actual operation effect of the green building is solved through analysis and identification. In this way, it is beneficial to move towards refined green building design and operation optimization. Therefore, it is very necessary to conduct long-term monitoring of the indoor and outdoor environment of the building, fully understand the mechanism, and then analyze the monitoring data and use relevant means to optimize the building environment [5]. IoT technology will reduce site injuries and make building construction more efficient. As the types of monitoring equipment become more and more complex, fieldbus technology can manage different types of monitoring equipment in a unified manner. At present, *RS-485* technology is mostly used to monitor the building environment and meteorological conditions, in order to stably and efficiently monitor the environment of the solar greenhouse in the northern cold region. In addition, *RS-485* can be used to establish a wired data transmission network for the acquisition node and data transmission between the acquisition node and the sensor group through the *Modbus* communication protocol and then use the 433 *MHz* wireless data acquisition device to connect with remote servers and clients [6–9].

**Figure 1:**
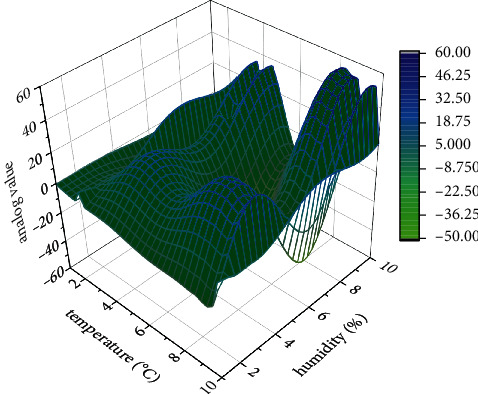
Hoflander's attitude change-persuasion model.

In this design system, another latest invention technology is used: wireless sensor network technology. Wireless sensor network technology has the advantages of convenience, flexibility, and wide application. Therefore, in recent years, many scholars have built relatively complete wireless sensor networks for environmental monitoring. With the help of wireless sensor network technology, that is, Arduino devices and *VOLTTRON* software platform to integrate *ZigBee* protocol sensors, a low-cost building performance monitoring system toolbox can be established. In addition, with the emergence of *ZigBee* protocol and Wi-Fi to form wireless sensor network technology, combined with the back-end monitoring information of *IoT* components, a monitoring system can be formed as an effective service platform [10]. From Figure 1, we can see that the types of building environment monitoring sensors mainly include temperature sensors, humidity sensors, formaldehyde sensors, haze sensors, *VOC* sensors, noise sensors, etc. Various types of sensors are usually composed of sensing modules, information processing modules, wireless communication modules, and energy supply modules [11]. The building energy consumption and environmental monitoring system based on the Internet of Things technology monitors the five energy systems of electricity, water, gas, heat consumption, and cooling consumption in the building energy consumption and analyzes the energy consumption data in combination with the environmental monitoring system inside the building. In the realization technology, the field bus instrument is used to realize the data acquisition of the 5 types of energy systems. Environmental parameter monitoring is implemented using ubiquitous wireless sensor network-based detectors. The construction industry is bringing real-time information into centuries-old processes. The devices and sensors of Internet of Things (IoT) are gathering workplace data in ways that are more affordable, efficient, and effective than previously thought.

**Figure 2:**
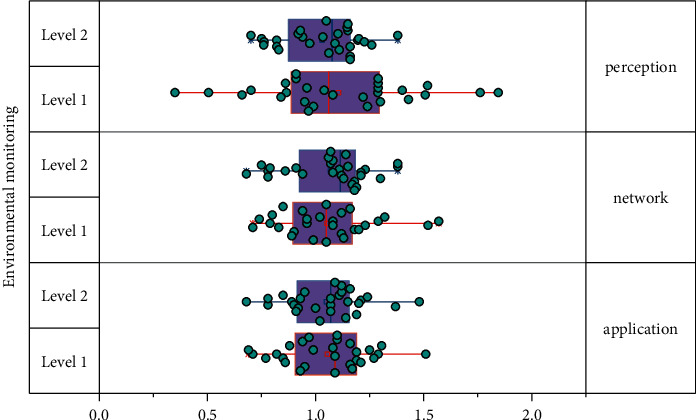
Calculation results of three levels of environmental monitoring.

Adopt industrial database and configuration software to realize the summarization and analysis of the collected data [12]. This system is based on computer, communication equipment, measurement, and control unit as basic tools and provides a basic platform for real-time data acquisition, switch status monitoring, and remote management and control of large public buildings. It can form any complex monitoring system with detection and control equipment [13]. The system mainly adopts a layered distributed computer network structure, which is generally divided into three layers: the station control management layer, the network communication layer, and the field device layer [14]. The field device layer is the data acquisition terminal, which is mainly composed of intelligent instruments. From Figure 2, we can see that the distributed *I/O* controller with high reliability and fieldbus connection is used to form the data acquisition terminal, and the stored building environment monitoring data is uploaded to the data center. Measuring instruments are responsible for the most basic data collection tasks, and the effective data monitored by them must be complete, accurate, and transmitted to the data center in real time.

## 2. The Composition and Principle of Radio Frequency Identification Technology

The composition of the Radio Frequency Identification (RFID) system includes four aspects:

1. Reader: It sends radio frequency signals to identify the electronic tag. After the identification is successful, it receives the information transmitted by the electronic tag and transmits it to the computer system for data analysis [15].
2. Electronic tag: It stores the relevant data information of the target to be identified. According to the energy source of the electronic tag, it can be divided into active tags, passive tags, and semiactive tags.
3. Antenna: including transmitting antenna and receiving antenna, which are used for energy and information transmission between readers and electronic tags and are an important part of RFID system. The quality of antenna performance directly affects the efficiency of energy transmission.
4. Computer system: data integration, processing, and analysis of the information received by the reader [16].

(1) $$\begin{array}{c} As=A{\left|C\right|}^{-\partial }. \end{array}$$

In the formula, *As* is the sensor resistance, *A* is a constant, and *C* is the gas concentration.

**Figure 3:**
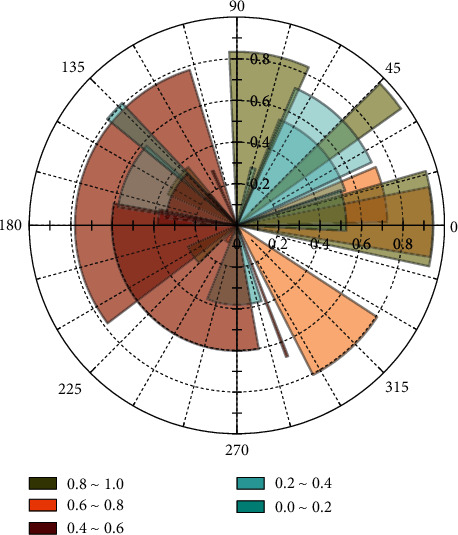
SVM training set classification accuracy.

The RFID system principle is composed of the following requirements. Firstly, a section of inquiry command is encoded, and the signal is modulated, and the radio frequency signal is sent to the electronic tag through the transmitting antenna, and finally the information stored by the tag is received by the receiving antenna [17]. This process enables the transfer of energy while simultaneously decoding, demodulating, and rectifying the received signal. From Figure 3, we can see that passive electronic tags are excited by energy, encode and modulate the information carried by themselves, and resend it to the reader, and the reader receives the information. Finally, the secondary information is submitted to the computer system for background processing, so as to complete the automatic identification work, and both modulation and demodulation are completed in the *RF* modem.

The transmission of signals between readers and electronic tags in an *RFID* system takes two types. The first is the inductive coupling model. This model is suitable for *RF* signals in the middle and low frequency bands. Similar to the transformer model, the magnetic induction loop of the electronic tag will be affected by the magnetic field change of the reader coil, which will generate an induced electromotive force and supply energy for the electronic tag, as shown in Figure 3, where the dotted box represents the magnetic field. The second is the electromagnetic backscattering model. This model is suitable for *RF* signals in the high frequency and microwave frequency bands. Compared with the previous model, due to the short wavelength of the radio frequency signal sent by the magic reader, after radiating to the electronic tag, part of it will carry the tag information and be reflected back to the reader. It is interesting that this model works similarly to the detection radar model [18].

(2) $$\begin{array}{c} f\left(x\right)=\text{sign}\left(\sum_{t=1}^{m}ay_{t}k\left(x_{t},x_{j}\right)+b\right). \end{array}$$

In the above formula, since the environmental data is nonlinear, the samples of the original input space should be mapped to the high-dimensional feature space, and then the optimal classification hyperplane should be constructed.

**Figure 4:**
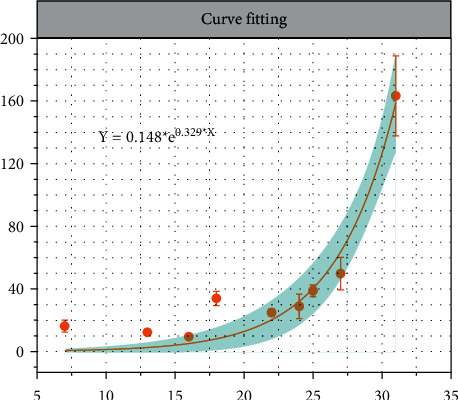
Simulated data fitting curve.

Compared with the traditional automatic identification technology, *RFID* technology has broad development prospects. People's research on sensors pays more attention to technology fusion. For example, by using the fusion of *RFID* technology and sensor technology, the sensor and the monitoring terminal can communicate and transmit information through radio frequency signals. From Figure 4, the data on the way accord with the relation of exponential function. From the distribution of fitting curve and actual data points, the mathematical model used in this study can accurately predict the trend of data development. This model can well achieve real-time monitoring and early warning of various indoor and outdoor toxic and harmful gas concentrations. In addition, it can effectively identify the concentration changes of some toxic and harmful gases over a period of time [19]. Based on the principle of this design procedure, the optimal allocation of energy can be effectively realized. In addition, the wireless networking mode and modular structure in the design are simple, the networking is convenient, and the construction and reconstruction are convenient. The visual interface provides a more convenient operation management mode for construction personnel, building management, and users and is easy to promote. This shows that the software can be used as an effective method for building green and energy-saving buildings [20].

## 3. Design of Automated Supervision Platform

### 3.1. Key Technologies of Automated Supervision Platform

**Figure 5:**
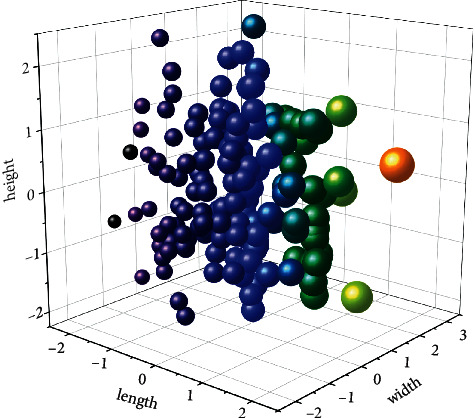
Motion recording of gas molecules in the environment.

First, establish the application and demonstration of environmental automation supervision on construction sites covering urban residential areas, and apply Internet of Things technology and cloud computing technology to the data center. From Figure 5, we can see that, after data analysis, statistics, spectrum analysis, storage, recording processing, and meteorological parameters, it is transmitted to the construction site project supervision command center, and various monitoring data are displayed in the real time at the construction site for the supervision of surrounding residents.

Second, establish a supervision cloud platform for environmental protection functional departments. The cloud platform mainly includes data communication supervision system, core management system, and data exchange platform. The core management system is mainly composed of a data display platform, a supervision platform, a public service platform for information release, and a standard data access platform. It is a bridge connecting front-end smart devices and data processing centers. The data processing center is mainly a bridge between the front-end intelligent monitoring sample collection equipment and the data processing center. The data center mainly has the management and control of the front-end intelligent monitoring sample collection equipment. The core management system has the function of virtualized management of all collected monitoring data. The data exchange platform saves the data to the corresponding data processing center according to the requirements of the cloud computing data storage platform.

Third, establish a unified data access standard. For example, the environmental information monitoring data and statistical information of construction companies have spatial attributes in addition to time and dynamics. It is suitable for expression using geographic information system. This system has a powerful data interface. The *GIS* system of the management department command center can integrate the construction site environmental data through the unified platform data access interface and dynamically and intuitively display the data of each environmental monitoring point.

### 3.2. Environmental Quality Detection and Feedback Circuit Design

#### 3.2.1. Sensor Module Circuit Design

**Figure 6:**
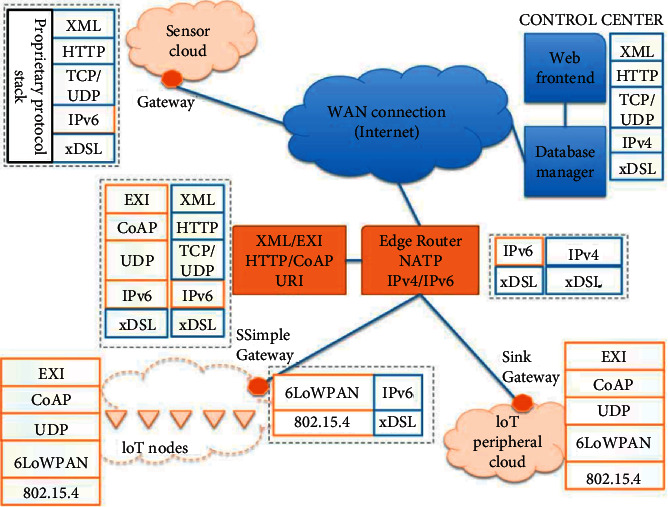
Conceptual image of a city IoT network.

The sensor used in the environmental quality detection part is composed of *GY-MCU680V1* sensor, *YW-51 GJ* sensor, *MQ-2* sensor, and *MQ-7* sensor. *GY-MCU680V1* indoor environmental quality sensor is a multisensor which is responsible for detecting air index, temperature, humidity, and air pressure. It integrates the BME680 four-in-one environmental sensor and processes the detected data through its own processor and outputs it through the serial port. *YW-51GJ* dust sensor is the sensor responsible for detecting *PM2.5* parameters. From Figure 6, we can see that the sensor uses the principle of scattering, by detecting the voltage value of the receiving element as a reference for the dust concentration. The sensor is integrated with an *ADC* converter and a processor, and the processor outputs the internal *AD*C conversion result through a serial port. *ZE08-CH2O* formaldehyde sensor is a sensor responsible for detecting formaldehyde concentration parameters. The sensor is responsible for measuring the formaldehyde concentration in the air and outputting it through the voltage value. The *MQ-2* and *MQ-7* sensors are hazardous gas sensors, which change the resistance of their gas-sensing resistors by reacting with the corresponding sensitive gases in the air, thereby producing voltage changes. When the voltage exceeds the limit, it will send out the corresponding high- and low-level signal through the *I/O* port to trigger the alarm.

#### 3.2.2. Display Module Circuit Design

The display module of the design system uses the 3.5-inch *TFT LCD* screen of Antike Technology which is matched with the core board. At the same time, the matching controller adopts *ILI9488* as the controller and uses the *FPC* cable to connect with the core board.

(3) $$\begin{array}{c} \text{PPD}=100-95\times e^{-0.03353\times PMV^{4}-02179\times PMV^{2}}. \end{array}$$

This formula expresses the calculation formula of environmental thermal comfort evaluation.

#### 3.2.3. Circuit Design of Alarm Module

The alarm module is *TELESKY* 3.3∼5V low-level drive active buzzer, of which *GND* and *VCC* are connected to the core board power supply circuit (3.3 V) to supply power to the module.

(4) $$\begin{array}{c} \text{APMV}=\frac{\text{pmv}}{\left(1+\lambda \times \text{pmv}\right)}. \end{array}$$

Among them, *λ* is the adaptive coefficient, which is -0.3 in the construction site.

#### 3.2.4. Module Circuit Design

Wi*-*Fi module uses Shenzhen Zhida *ESP8266* Wi*-*Fi module, which uses *ESP8266* processor to handle Wi-Fi corresponding operations, and uses serial port to control the processor. The *ZigBee* module uses the Deep Link Innovation *DL-LN33* ZigBee module, which uses the *CC2530* processor to handle the corresponding operations of the *ZigBee*, and uses the serial port to control the processor.

(5) $$\begin{array}{c} y=\frac{2\left(X_{t}-X_{\min}\right)}{X_{\max}-X_{\min}}-1. \end{array}$$

In the formula, *x* represents the collected air quality data, and *y* represents the data after normalization and transformation, which can reduce the deviation between the result and the actual value.

(6) $$\begin{array}{c} n=\sqrt{i+o}+m. \end{array}$$

In the formula, *n*, *i*, and *o*, respectively, represent the number of monitoring points, the number of calculation units, and the number of display units.

### 3.3. Overall System Scheme and Basic Software and Hardware Design

**Figure 7:**
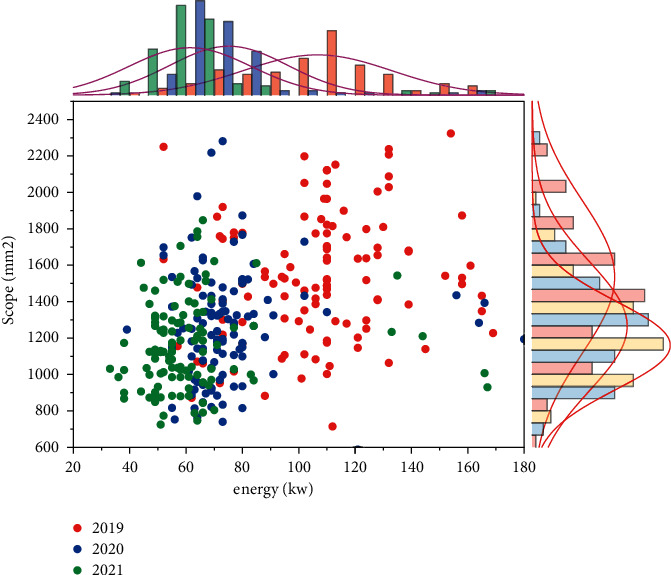
Harmful gas movement range and release energy.

From Figure 7, we can see that the building environment monitoring system mainly includes three modules: the collection and control module of environmental parameter information, the information management center module, and the host computer or user terminal module.

1. The collection and control module of environmental information adopts *ZigBee* technology to form a wireless communication network. The *ZigBee* terminal node is connected to a sensor that collects environmental information and a controller that adjusts and controls the environment. The sensor periodically collects environmental data. The *ZigBee* terminal node sends the collected environmental data to the *ZigBee* coordinator node, which is responsible for collecting the environmental data collected by the terminal node, and then transmits it to the *ARM* home gateway. At the same time, the coordinator node is also responsible for forwarding the control instructions of the home gateway to the terminal node to realize the process of automatic control.
2. The information management center module uses the *ARM* embedded server as the home gateway, stores the environmental parameters in the database, processes and analyzes the collected environmental information according to the preset environmental parameters, and automatically sends the control instructions to the corresponding front-end controller. *ARM* embedded low-power and high-performance features make the system more secure, reliable, and energy efficient.
3. User terminals mainly include communication equipment such as smart phones, tablet computers, and *PCs.* These devices can be directly connected to the gateway through Wi-Fi indoors, view environmental parameters, and send operating instructions, while outdoors they need to be remotely controlled through a *WEB* server.

**Figure 8:**
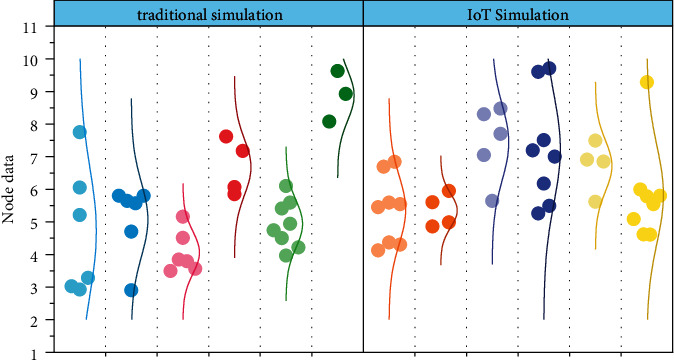
Various parameter values for the two algorithms.

In order to comprehensively monitor environmental information, the system detects and controls environmental parameters as follows: Temperature and humidity are the most sensitive factors affecting human comfort. The main components of household gas carbon monoxide and formaldehyde gas produced by furniture and decoration are common indoor pollutant gases, which have great harm to people's health. When the set threshold is exceeded, the system maintains air circulation by controlling the ventilation equipment and activates the alarm device (Figure 8). Fire and illegal intrusion are related to the safety of people's lives and properties. When a fire information or illegal intrusion event is detected, the system will immediately activate the fire or security alarm system.

As the core of the whole system, the home gateway and project center process the environmental data and judge the control mode after reading the environmental data. If it is in the manual control mode, it will enter the manual control strategy, and carry out environmental control according to the user's control instructions. If it is an automatic control mode, the judgment of the scene mode is carried out according to the data of the environment, and each scene mode will correspond to its corresponding control strategy and store and display the data.

According to the characteristics of the intelligent environmental monitoring system, the principle of modularity should be adopted in the design of the system, so that each module has its independence. In this way, when the system adds new modules or exits old modules, the system will remain stable. At the same time, since each sensor node needs to work for a long time, it is necessary to reduce the power consumption of the node as much as possible. In order to reliably realize the functions of the intelligent environmental monitoring system, it is necessary to design from two aspects of the system's hardware and system's software.

## 4. System Hardware Design

The system uses *ZigBee* technology to monitor the environmental parameters of the building site. First, use the terminal sensor node to collect environmental data, and then send it to the coordinator terminal node through *ZigBee* two-way wireless communication, upload it to the remote client, and display it in real time through the host computer. The user can monitor the site environment in real time through the host computer, and if an abnormality is found, the alarm can be timely and the intelligent equipment inside the building can be reversely controlled to deal with the dangerous situation. In order to facilitate the early detection of abnormalities by the personnel in the building, relevant environmental parameters will be directly displayed through the *LCD* liquid crystal display installed on the coordinator node.

(7) $$\begin{array}{c} V_{ij}=X_{ij}+\text{rand}\left(-1,1\right)\left(X_{ij}-X_{kj}\right). \end{array}$$

Among them, *i* and *j* represent the objective function value of the *i*th node, and *k* is a random number.

ZigBee technology is one of the wireless communication technologies. Compared with wired transmission, it has great advantages in construction sites with high security requirements. It has the advantages of low-power consumption, low cost, short distance, low complexity, and fast connection speed. In addition, *ZigBee* communication connection takes only 25 ms, while other wireless communication technologies such as *Bluetooth* require 5 s and Wi-Fi takes 11 s. In the face of frequent environmental data transmission, *ZigBee* is more in line with the project requirements.

(8) $$\begin{array}{c} P_{i}=\frac{f\left(X_{i}\right)}{\sum_{n=1}^{N_{s}}f\left(X_{n}\right)}, \end{array}$$

where *f(x)* is the software and environment fitness value.

In this design, a single-chip integrated *SoC* solution is mainly used, which is mature, small in size, and convenient and flexible to use. The *ZigBee* hardware platform selects the *CC2530* chip produced by *TI*. Although the sleep voltage of the chip is not the highest, its transmit power and receive sensitivity are the highest, reaching 4.5 dBm and −97 dBm, respectively.

(9) $$\begin{array}{c} Y=Hx+{\left[e_{1},e_{2},e_{3},\ldots ,e_{n}\right]}^{T}. \end{array}$$

With the ambient noise and the sensor noise, the actual measured value of the system is *x*.

This system needs to realize real-time data uploading to the cloud, so the system chooses the *ESP-12F* Wi-Fi module developed by Anxinke Technology. The module is inexpensive and mature in technology, with an operating temperature range of −40 to 125°C. The temperature setting can ensure the stability of the environment in the construction site, with low standby power and fast wake-up speed, which can meet the requirements of real-time data transmission.

(10) $$\begin{array}{c} \hat{X}={\left(H^{T}WH\right)}^{-1}H^{T}WY, \end{array}$$

where *W* is a positive definite diagonal weighting matrix.

Use *DHT11* sensor to collect warehouse temperature and humidity data. *DHT11* collects and combines temperature and humidity data, outputs it in the form of digital signal, and uses special analog signal acquisition, conversion technology, and temperature and humidity sensing technology to ensure that the sensor has good stability and reliability. The average working current of *DHT11* does not exceed 0.5 mA, the power consumption is low, and the working voltage range is 3∼5.5 V, which matches the power supply equipped in this design.

## 5. System Software Design

**Figure 9:**
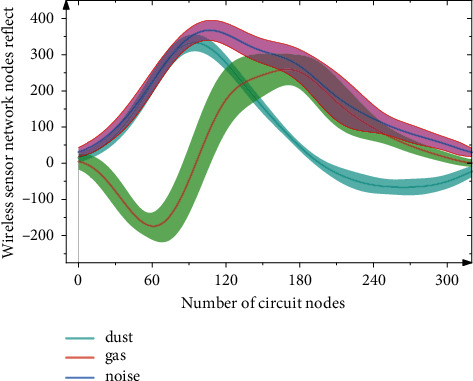
Wireless sensor circuit network node unit diagram.

Sensor-related operations include initializing the sensor, reading sensor values, and writing data to an array in a fixed format. During initialization, it is only necessary to open the clock of the corresponding port and initialize the *GPIO*. The initialization of the environmental quality detection sensor and the *PM2.5* sensor needs to initialize the corresponding *UART.* The former baud rate is 9600; the latter is 2400. From Figure 9, when the initialization is completed, the *UART* needs to be enabled, the Interrupt Enable is turned on, and the first data acquisition after power-on is performed. Finally, the obtained data is filled into the corresponding array according to the node type, and the reading of the environmental quality monitoring sensor refers to the fixed data format.

Wi-Fi mainly includes operations such as initializing the module, sending basic commands, and sending and receiving messages. This system uses *ESP8266* chip for network operation; the chip can set up *TCP/IP* server and monitor automatically through serial port commands. If a remote connection is established with the chip through *Socket* communication, the server will automatically receive this request and automatically assign a source *ID* for communication with the current connection. After the connection is established, if there is still no actual communication content after a certain period of time, the connection will be automatically disconnected to prevent the connection line from being occupied for a long time.

(11) $$\begin{array}{c} E\left[{\left(x-\hat{x}\right)}^{2}\right]=E\left\{\sum_{i=1}^{n}{\left[\left(\frac{w_{i}}{\sum_{i=1}^{n}w_{i}}\right)\right]}^{2}\varepsilon_{i}^{2}\right\}. \end{array}$$

The above formula represents the sum of the squares of the estimation errors of *x*.

**Figure 10:**
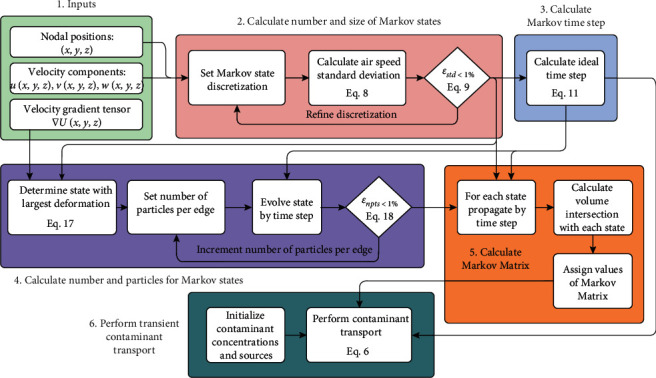
Method from input to accurate matrix of contaminant transport.

Select a residential building as an example in the design, import the model into the Unity engine, and add text boxes for displaying data. As Figure 10 shows, a text box for displaying data related to environmental quality is preset on the user form in advance, and the text box on the interface is bound to the corresponding control variable in the main program through the object binding process. When the application is running, a query command is sent to the designated environmental quality monitoring node server, and the text box is filled by parsing the returned data; that is, real-time data embedding is achieved.

*UML* provides a variety of model diagrams. In software development, these model diagrams can be used to describe the work in each stage of development, which is convenient for work implementation and personnel communication. In real-world applications, *UML* is mainly used in two phases: requirement analysis and system design. In the requirements analysis stage, the use case model diagram is mainly used to describe the system requirements more intuitively and facilitate communication with users. In the design phase of the system, the static model and dynamic model of the system are mainly used.

(12) $$\begin{array}{c} \varepsilon_{mi}^{2}=E{\left(y_{mi}-\bar{y_{m}}\right)}^{2}, \end{array}$$

where *ε* is the measurement variance of the mth measurement from the ith sensor.

## 6. System Testing and Evaluation

In order to test the function of the system, temperature and humidity, harmful gases, and illegal intrusion are used as test samples, so that different environmental areas in the building can be simulated. The ambient temperature and humidity are detected by the *DHT11* temperature and humidity sensor with strong anti-interference ability. Hazardous gas sensors use *MQ-5* semiconductor combustible gas sensors with high sensitivity, and *HC-SR501* human body infrared sensors are used to monitor illegal intrusion events. The biggest advantage of smart site construction project construction is that the site staff can remove faults in time and reduce the occurrence of engineering project safety accidents.

Start each module of the system, the environmental data acquisition system will send the network access request to the gateway through the *ZigBee* wireless sensor network, and the gateway will send the command to assign *ID* after receiving the request instruction. If the light-emitting diode of the environmental data acquisition system flashes three times at this time, it means that the environmental data acquisition system is successfully connected to the network. In this test, the on/off control of the relay is simulated by the brightness of the *LED* light.

Compared with the traditional environmental monitoring system, this system has the following advantages:

1. Simple and scalable. There is no need to specially set up independent systems for different objects. In this system, multiple different environmental objects can be monitored, transmitted, and processed in the same network.
2. The accuracy of the data is high. In the *ZigBee* network, the adopted intelligent optimization algorithm makes comprehensive decision-making, which avoids the error caused by monitoring a single node, and can greatly improve the accuracy of data monitoring.
3. The node operation is flexible. Users can add environmental information collection nodes or reduce collection nodes according to their own needs, without requiring additional wiring, and the nodes are flexible in operation and can be placed freely.

## 7. Conclusion

In this paper, through the analysis of the research status of environmental monitoring system design and application scheme, a set of integrated real-time monitoring system based on Internet of Things technology has been successfully constructed, and it is truly stable in the environmental monitoring system, with physical measurement experiments for indoor and outdoor environments of buildings. Based on the scalability and wide application of the system, scholars in the related fields of construction engineering science in the future can expand as needed to meet the comprehensive real-time monitoring of various building types. The platform connects to the Internet through Wi*-*Fi wireless network for data communication, which can realize office or home networking, and real-time sharing of monitoring data from multiple monitoring points. The access and setting of the equipment can be completed through the computer or mobile *APP*, and the display software is used to realize the three-dimensional visualization of the indoor environmental quality parameter information in the building model. Internet of Things technology has been continuously accumulated and upgraded, and the industrial chain has gradually improved and matured. IoT technology will bring opportunities to upgrade infrastructure construction around the world.

## Data Availability

The data used to support the findings of this study are available from the corresponding author upon request.

## References

[1] Esteves A., Esteves M. J., Mercado M. V., Barea G., Gelardi D. (2018). Building shape that promotes sustainable architecture. Evaluation of the indicative factors and its relation with the construction costs. *Architecture Research*.

[2] Marques G., Pitarma R. (2016). An indoor monitoring system for ambient assisted living based on internet of Things architecture. *International Journal of Environmental Research and Public Health*.

[3] Aksoezen M., Daniel M., Hassler U., Kohler N. (2015). Building age as an indicator for energy consumption. *Energy and Buildings*.

[4] Ala G., Di Filippo G., Viola F. (2020). Different scenarios of electric mobility: current situation and possible future developments of fuel cell vehicles in Italy. *Sustainability*.

[5] Antoniadis C. N., Martinopoulos G. (2019). Optimization of a building integrated solar thermal system with seasonal storage using TRNSYS. *Renewable Energy*.

[6] Ge M., Bangui H., Buhnova B. (2018). Big data for internet of Things: a survey. *Future Generation Computer Systems*.

[7] Andronie M., Lăzăroiu G., Iatagan M., Uță C., Ştefănescu R., Cocoșatu M. (2021). Artificial intelligence-based decision-making algorithms, internet of Things sensing networks, and Deep learning-assisted smart process management in cyber-physical production systems. *Electronics*.

[8] Arslan G. (2021). Loneliness, college belongingness, subjective vitality, and psychological adjustment during coronavirus pandemic: development of the College Belongingness Questionnaire. *Journal of Positive School Psychology*.

[9] Ng C. K., Wu C. H., Yung K. L., Ip W. H., Cheung T. (2018). A semantic similarity analysis of Internet of Things. *Enterprise Information Systems*.

[10] Lei L., Tan Y., Zheng K., Liu S., Zhang K., Shen X. (2020). Deep reinforcement learning for autonomous internet of Things: model, applications and challenges. *IEEE Communications Surveys & Tutorials*.

[11] Harrer M., Adam S. H., Fleischmann R. J. (2018). Effectiveness of an internet-and app-based intervention for college students with elevated stress: randomized controlled trial. *Journal of Medical Internet Research*.

[12] Rodrigues J. J. P. C., De Rezende Segundo D. B., Junqueira H. A. (2018). Enabling technologies for the internet of health Things. *Ieee Access*.

[13] Limones-Rodríguez N., Marzo-Artigas J., Pita-Lopez M. F., Díaz-Cuevas M. P. (2018). The impact of climate change on air conditioning requirements in Andalusia at a detailed scale. *Theoretical and Applied Climatology*.

[14] Lolli N., Hestnes A. G. (2014). The influence of different electricity-to-emissions conversion factors on the choice of insulation materials. *Energy and Buildings*.

[15] López-Ochoa L. M., Las-Heras-Casas J., López-González L. M., García-Lozano C. (2017). Environmental and energy impact of the EPBD in residential buildings in cold Mediterranean zones: the case of Spain. *Energy and Buildings*.

[16] De Luca G., Ballarini I., Lorenzati A., Corrado V. (2020). Renovation of a social house into a NZEB: use of renewable energy sources and economic implications. *Renewable Energy*.

[17] Grimmelikhuijsen S., Jilke S., Olsen A. L., Tummers L. (2017). Behavioral public administration: combining insights from public administration and psychology. *Public Administration Review*.

[18] Zilio D. (2016). On the autonomy of psychology from neuroscience: a case study of skinner’s radical behaviorism and behavior analysis. *Review of General Psychology*.

[19] Wood W., Rünger D. (2016). Psychology of habit. *Annual Review of Psychology*.

[20] Sheeran P., Bosch J. A., Crombez G. (2016). Implicit processes in health psychology: diversity and promise. *Health Psychology*.

